# Fermentative hydrogen production from Jerusalem artichoke by *Clostridium tyrobutyricum* expressing exo-inulinase gene

**DOI:** 10.1038/s41598-017-07207-7

**Published:** 2017-08-11

**Authors:** Ling Jiang, Qian Wu, Qing Xu, Liying Zhu, He Huang

**Affiliations:** 1 College of Food Science and Light Industry, Nanjing Tech University, Nanjing, 210009 People’s Republic of China; 2 College of Biotechnology and Pharmaceutical Engineering, Nanjing Tech University, Nanjing, 210009 People’s Republic of China; 3 College of Chemical and Molecular Engineering, Nanjing Tech University, Nanjing, 210009 People’s Republic of China; 4 College of Pharmaceutical Sciences, Nanjing Tech University, Nanjing, 210009 People’s Republic of China

## Abstract

*Clostridium tyrobutyricum* ATCC25755 has been reported as being able to produce significant quantities of hydrogen. In this study, the exo-inulinase encoding gene cloned from *Paenibacillus polymyxa* SC-2 was into the expression plasmid pSY6 and expressed in the cells of *C*. *tyrobutyricum*. The engineered *C*. *tyrobutyricum* strain efficiently fermented the inulin-type carbohydrates from Jerusalem artichoke, without any pretreatment being necessary for the production of hydrogen. A comparatively high hydrogen yield (3.7 mol/mol inulin-type sugar) was achieved after 96 h in a batch process with simultaneous saccharification and fermentation (SSF), with an overall volumetric productivity rate of 620 ± 60 mL/h/L when the initial total sugar concentration of the inulin extract was increased to 100 g/L. Synthesis of inulinase in the batch SSF culture was closely associated with strain growth until the end of the exponential phase, reaching a maximum activity of 28.4 ± 0.26 U/mL. The overall results show that the highly productive and abundant biomass crop Jerusalem artichoke can be a good substrate for hydrogen production, and that the application of batch SSF for its conversion has the potential to become a cost-effective process in the near future.

## Introduction

Hydrogen production technologies have received growing research attention all over the world in recent years, due to the significant increase in hydrogen demand as an alternative energy source as well as a feedstock for the production of chemicals and food-oil industries. Nowadays, approximately 90% of the current hydrogen production is obtained by the conversion of fossil fuels, which requires a mass of energy and emits huge greenhouse gasses^1^. In contrast to chemical methods, biological processes using carbohydrate-based substrates for hydrogen production, such as indirect photolysis, photo-fermentation, and dark fermentation, are economical and environmentally friendly^2, 3^. Extensive researches in the past two decades have been focused on the promising route of biohydrogen production via dark fermentation using pure strains in batch and continuous systems, which can be operated at ambient temperature and pressure with minimal energy requirements^4^. *Clostridia* are the most significant microorganisms in anaerobic hydrogen fermentation^5^. So far, a wide range of organic compounds, such as glucose, xylose, ribose, glycerol, hydrolysates of diverse starch sources, and sugar beet molasses, have been used as substrates for hydrogen fermentation using *Clostridium* species (Table 1). In general, the carbon source is usually used in relatively high concentrations compared with other media components, and thus contributes most to the total cost of raw materials. Considering the cost-efficiency of fermentative hydrogen production, the exploitation of non-food residues originating from agro-industrial activities as carbon sources has therefore been strongly stimulated.

**Table 1:** Summary of hydrogen fermentation with Clostridium species.

*Clostridium* species	Substrates profile	Yield of hydrogen (mol/mol glucose)	Reference
*C*. *tyrobutyricum* ATCC 25755 wild type	glucose	2.61	33
*C*. *tyrobutyricum* ATCC 25755 DG-8	cassava starch	3.20	28
*C*. *tyrobutyricum* ATCC 25755 (△*ptb*)	glucose and xylose	2.34	34
*C*. *tyrobutyricum* FYa102	glucose	1.47	35
*C*. *tyrobutyricum* MPP-41 (DQ911273)	glucose	1.96	36
*C*. *tyrobutyricum* JM1	glucose	1.79	37
*C*. *butyricum* IFO13949	sweet potato starch	2.40	38
*C*. *butyricum* CWBI1009	glucose	1.7	39
*C*. *butyricum* DSM 10702	starch	2.74	40
*C*. *acetobutylicum* ATCC 824	glucose	3.01	41
*C*. *acetobutylicum* DSM 792	beet molasses	2.80	42
*C*. *beijerinkii* DSM 791	wheat starch	0.60	43
*C*. *beijerinckii* DSM 1820	glucose	2.91	44
*C*. *beijerinckii* YA001	xylose	2.31	45
*C*. *pasteurianum*	crude glycerol	0.63	46
*Clostridium sp*. 6A-5	glucose	0.68	47
*Clostridium sp*. YM1	xylose	0.82	48
*Clostridium sp*. IBUN	glycerol	0.20	49
*Clostridium sp*. PROH2	ribose	1.23	50

**Figure 1:**
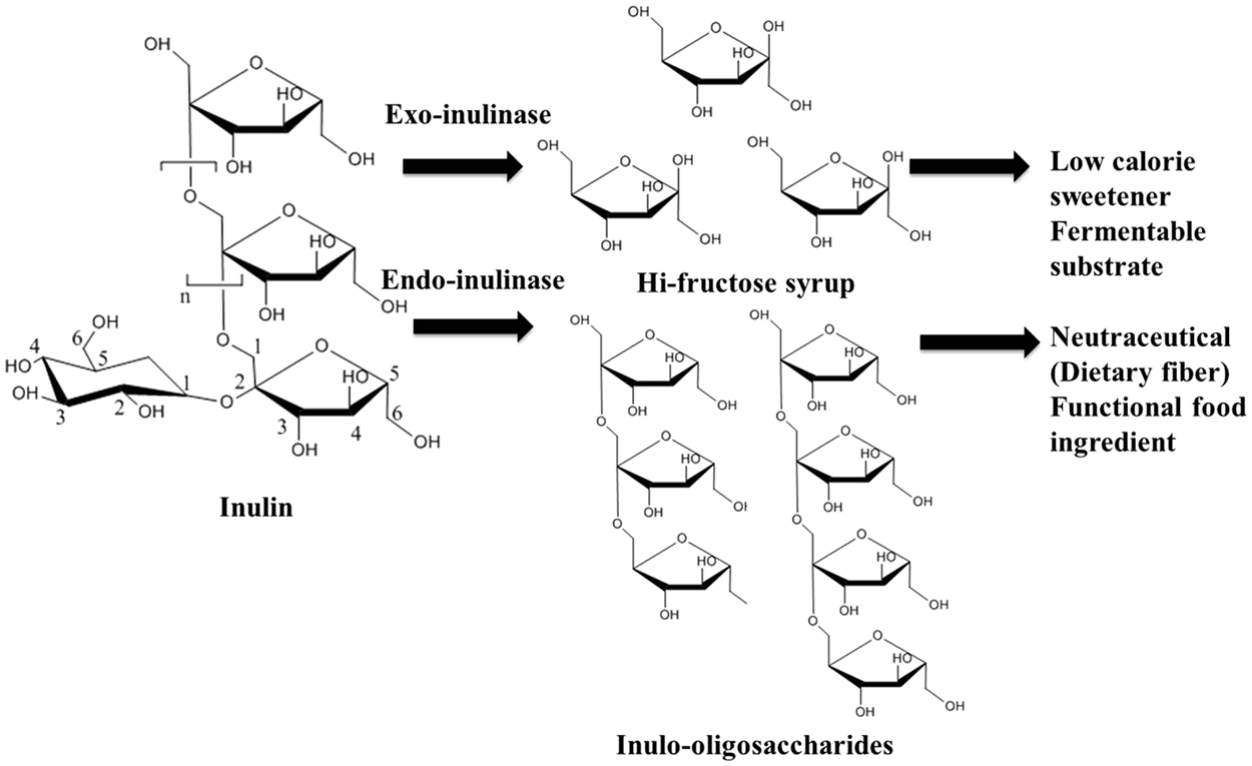
Hypothetical inulin being acted upon by microbial exo- and endo-inulinase enzymes. Action of exo-inulinase releases fructose from the macromolecule while endo-inulinase produces inulooligosaccharides.

Jerusalem artichoke is one of the least expensive and most widely available non-grain crops^6^. It shows high resistance to frost and various plant diseases, which resulted in its wide cultivation in northern China for environmental protection^6^. Fresh Jerusalem artichoke tubers are rich in carbohydrates, of which 70–90% (w/w) is inulin. Moreover, Jerusalem artichoke has been widely utilized as a biotechnological feedstock in recent years, including in the microbial production of lactic acid^7, 8^, propionic acid^9^, butyric acid^10^, ethanol^11^, 2,3-butanediol^12, 13^, lipids^14^ as well as single-cell protein^15^. However, because most wild-type microorganisms cannot secrete inulinase, an acidic or enzymatic hydrolysis pretreatment of the inulin was traditionally required prior to fermentation, which significantly increases the cost of the process^11^. Inulin consists of linear chains of β-2,1-linked D-fructofuranose molecules terminated by a glucose residue through a sucrose-type linkage at the reducing end. Inulinases are fructofuranosyl hydrolases, the general reaction of which mainly involves the action of two enzymes: exo-inulinase (EC 3.8.1.80) and endo-inulinase (EC 3.2.1.7). Exo-inulinases can be used for the production of high-fructose syrup from natural inulin (saccharification), while endo-inulinases can be used for the production of fructooligosaccharides of varying lengths (Fig. 1).

*Paenibacillus polymyxa* (formerly *Bacillus polymyxa*) is one of the few bacteria known to be able to produce inulinases and ferment inulin into valuable chemicals without previous hydrolysis^16^. In the present study, the gene encoding exo-inulinase from *P*. *polymyxa* SC-2 was cloned into *Clostridium tyrobutyricum* ATCC 25755, which enabled the host bacterium to efficiently convert inulin from Jerusalem artichokes into hydrogen. Further experiments were conducted to develop a simultaneous saccharification and fermentation (SSF) process for hydrogen production from inulin utilizing this heterologous inulinase in *C*. *tyrobutyricum*.

## Experimental details

### Plasmids, strains and cultivation

The plasmids and strains used in the present study are listed in Table 2. *P*. *polymyxa* SC-2 was isolated from the rhizosphere of pepper in Guizhou, China^17^. It was cultivated in 10 mL of selective medium (30.0 g/L inulin, 1.0 g/L (NH_4_)_2_HPO_4_, 0.50 g/L MgSO_4_·7H_2_O, 1.0 g/L NaCl, 2.0 g/L (NH_4_)_2_SO_4_, pH 6.0) and cultured at 30 °C and 120 rpm for 2 days. The host strain *E*. *coli* TOP10 was obtained from Novagen and grown in Luria-Bertani (LB) medium with 100 μg/mL ampicillin added if necessary. *C*. *tyrobutyricum* ATCC 25755 (purchased from Guangdong culture collection center, collection number: GIM 1.262) was used as the host strain. The cultivation medium was composed of 5 g/L yeast extract, 5 g/L peptone 3 g/L (NH_4_)_2_SO_4_, 1.5 g/L K_2_HPO_4_, 0.6 g/L MgSO_4_·7H_2_O, 0.03 g/L FeSO_4_·7H_2_O, and was sterilized by autoclaving at 121 °C for 20 min as reported previously^18^. In order to promote plasmid retention in *C*. *tyrobutyricum*, 25 μg/mL of erythromycin was added to the cultivation medium. The batch SSF cultivations were performed in a 5-L stirred-tank fermentor with a working volume of 2 L, as well as a 500-L stirred-tank fermentor with a working volume of 200 L. The fermentors were operated at 37 °C, agitated at 150 rpm, and the pH was controlled at 6.0 by adding 5 mol/L NaOH solution facilitated by an on-line sensing and dosing system. Anaerobiosis was maintained by sparging the medium with N_2_ (10 mL/min) for 60 min as previously reported^19^.

**Table 2:** Strains and plasmids used in this study.

Strains and Plasmids	characteristic	Source or reference
*Paenibacillus polymyxa* SC-2	wild type	17
*Clostridium tyrobutyricum* ATCC 25755	wild type	ATCC
*E*. *coli* DH5α	competent cells	Vazyme
*E*. *coli* TOP10	carries the pAN2 vector	Invitrogen
pAN2	methylated vector, *Φ3TI*, *pl5A ori*, Tet^R^	51, 52
pSY6	general vector for *Clostridium sp*	53
pSY6-*INU*	recombinant plasmid	This study
Mutant strain	*Clostridium* strain with introduced *INU* gene	This study

### Construction of the expression vector carrying the exo-inulinase gene and transformation of *C*. *tyrobutyricum*

Plasmid pSY6 with *lac* promoter was used as the vector backbone for cloning^20^. *E*. *coli* DH5α was used for vector construction. The *inu* gene encoding exo-inulinase was amplified by PCR using the genomic DNA of *P*. *polymyxa* SC-2 strain as template with the primers F: 5′-CCGCTCGAGATGAACGTTGTTAGGCAAGAG AAAT-3′ and R: 5′-CGCTGTACATCATTTCAGCGCATAAAGCTCCAGC-3′ (the bases underlined are the recognition sites of restriction enzymes *Xho*I and *BsrG*I)^17^. The PCR product was cloned into the *Xho*I and *BsrG*I sites of plasmid pSY6, supplemented with ampicillin (100 μg/mL), yielding exo-inulinase expression recombinant plasmid pSY6-*inu*, then methylated in *E*. *coli* TOP10 (pAN2)^21^. The recombinant plasmids extracted from the positive transformants were digested with *BgI*II. After verification by DNA sequencing (GENEWIZ, China), the recombinant plasmid was used for the electrotransformation of *C*. *tyrobutyricum* (Fig. S1). Plasmid pSY6 without the insert was used as the empty vector control. After cultivation on agar plates with 25 μg/mL of erythromycin for 2 days, positive transformants were selected at random, used to inoculate the inulinase production medium in replicates, and cultivated at 37 °C for 3 days^22^. The cultures were centrifuged at 12,000× *g* and 4 °C, and the inulinase activity in the supernatants of different transformants was determined as described below.

### Preparation of inulin extracts from Jerusalem artichoke

Jerusalem artichoke tubers were purchased from a local market (Nanjing, Jiangsu, China) during the harvest season from October to November 2015. About 200 g of the Jerusalem artichoke tubers were washed, peeled, smashed, suspended in 500 mL of distilled water and cooked at 100 °C for 5 min. The inulin extract was filtered under vacuum and the total sugar concentration in the filtrate was adjusted to 20 g per 100 mL of the supernatant, which was finally autoclaved at 121 °C for 20 min.

### Analysis methods

For protein expression analysis, cell-free extracts (CFE) were mixed with an equal amount of 3-fold concentrated loading buffer (10 mmol/L Tris-HCl pH 6.8, 4% (w/v) sodium dodecyl sulfate, 20% (v/v) glycine, 0.2% bromophenol blue, 2% (v/v) 2-mercaptoethanol). After boiling for 10 min, 10 µl of each sample was analyzed by sodium dodecyl sulfate polyacrylamide gel electrophoresis (SDS-PAGE). Protein concentrations were determined by the method of Bradford using bovine serum albumin as a standard. Cell concentrations were analyzed by measuring the optical density of the cell suspension at OD_600_ with a spectrophotometer (Ultrospec 3300 pro, Amersham Bioscience). Quantitative analysis of acids (butyric and acetic acid) and reducing sugars (glucose and fructose) was performed by HPLC (Agilent 1100, Agilent Technologies) equipped with an HPX 87H column (Bio-Rad, Hercules, CA, USA) and a refractive index detector (Agilent1100, G1362A)^23^. The H_2_ and CO_2_ concentrations in the exhaust gas were determined using the PGM 54 MultiRAE IR gas monitor (RAE system Inc., San Jose, USA).

The inulinase activity was assayed by the method of Mughal *et al*.^24^. A reaction mixture containing 0.1 mL of the enzyme extract (the supernatant obtained earlier) and 0.9 mL of a sodium acetate buffer (0.1 M, pH 5.5) containing 2% (w/v) inulin was incubated at 50 °C for 15 min. The enzyme was inactivated by keeping the reaction mixture at 90 °C for 10 min. The amount of reducing sugars in the reaction mixture was assayed by the Nelson–Somogyi method^25^. One unit of inulinase activity is defined as the amount of reducing sugar produced (μmol) per minute under the assay conditions used in this study. All results were obtained from the means of triplicate assays.

## Results and Discussion

### Expression of exo-inulinase in *C*. *tyrobutyricum*

**Figure 2:**
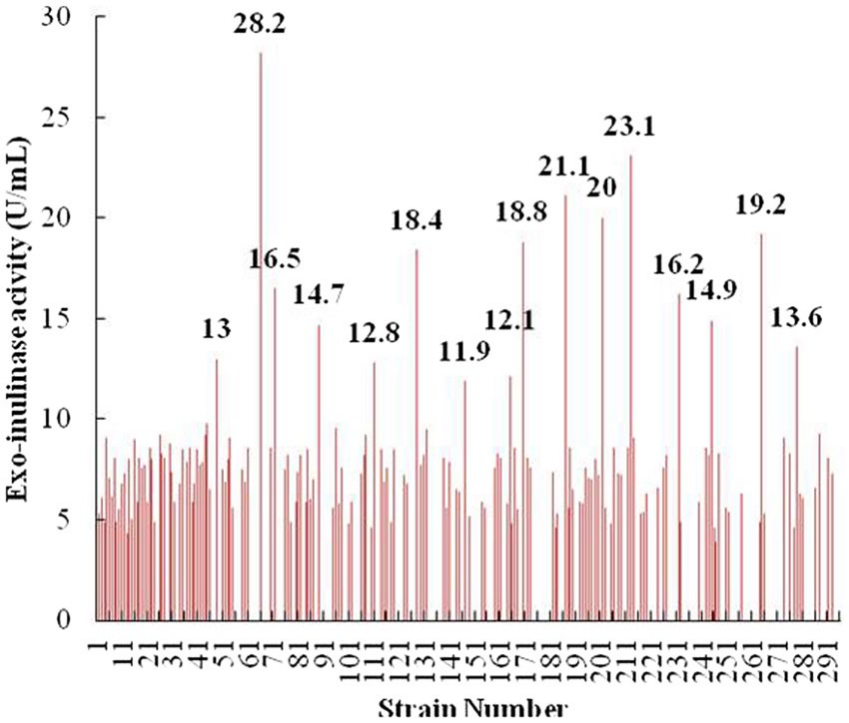
Inulinase activities of different transformants carrying the exo-inulinase gene. Numerical values in the figure are the activities of exo-inulinase from the 16 different transformers.

It has already been known from previous whole-genome sequencing studies that wild-type *C*. *tyrobutyricum* cannot secrete inulinase^26^. In order to make the engineered *C*. *tyrobutyricum* hydrolyze inulin so that it can produce hydrogen directly without the need for a separate inulin hydrolysis process, the exo-inulinase gene cloned from *P*. *polymyxa* SC-2 was ligated into the expression vector pSY6, and then transformed into *C*. *tyrobutyricum* after being methylated in *E*. *coli* TOP10 (pAN2). After DNA transformation, some 300*C*. *tyrobutyricum* mutant colonies were chosen from the agar plates and the inulinase activities in the supernatants were examined to ensure that they were positive transformants. It was revealed that most of the clones had inulinase activities lower than 10.0 U/mL. Furthermore, there was a 46.3% false-positive rate (139 out of the 300 clones shown in Fig. 2). A relatively high false-positive rate, which was further illustrated by colony PCR identifications not yielding the target band (data not shown), may be partly due to the low transformation efficiency in the genus *clostridia*. It is generally acknowledged that genetic engineering of the genus *clostridia* has been developed slowly compared with that of other bacteria, one of the major reasons might be the restriction and modification system which degrades foreign DNA^20^. However, the transformant J66 was showed the highest activity of 28.2 U/mL among the top 16 values (Table 3). As expected, the wild-type strain *C*. *tyrobutyricum* had no inulinase activity as shown in Table 3, which was in accordance with the genome sequencing result^26^.

**Table 3:** Exo-inulinase activities of the different transformants.

Transformants of *C*. *tyrobutyricum*	Exo-inulinase activity (U/mL)
J49	13.0 ± 0.35
J66	28.2 ± 1.36
J72	16.5 ± 0.29
J89	14.7 ± 0.42
J111	12.8 ± 0.33
J128	18.4 ± 0.58
J147	11.9 ± 0.62
J165	12.1 ± 0.48
J170	18.8 ± 0.87
J187	21.1 ± 1.01
J202	20.0 ± 0.98
J213	23.1 ± 1.23
J232	16.2 ± 0.78
J245	14.9 ± 0.55
J265	19.2 ± 0.66
J279	13.6 ± 0.79
Wild-type	0

**Figure 3:**
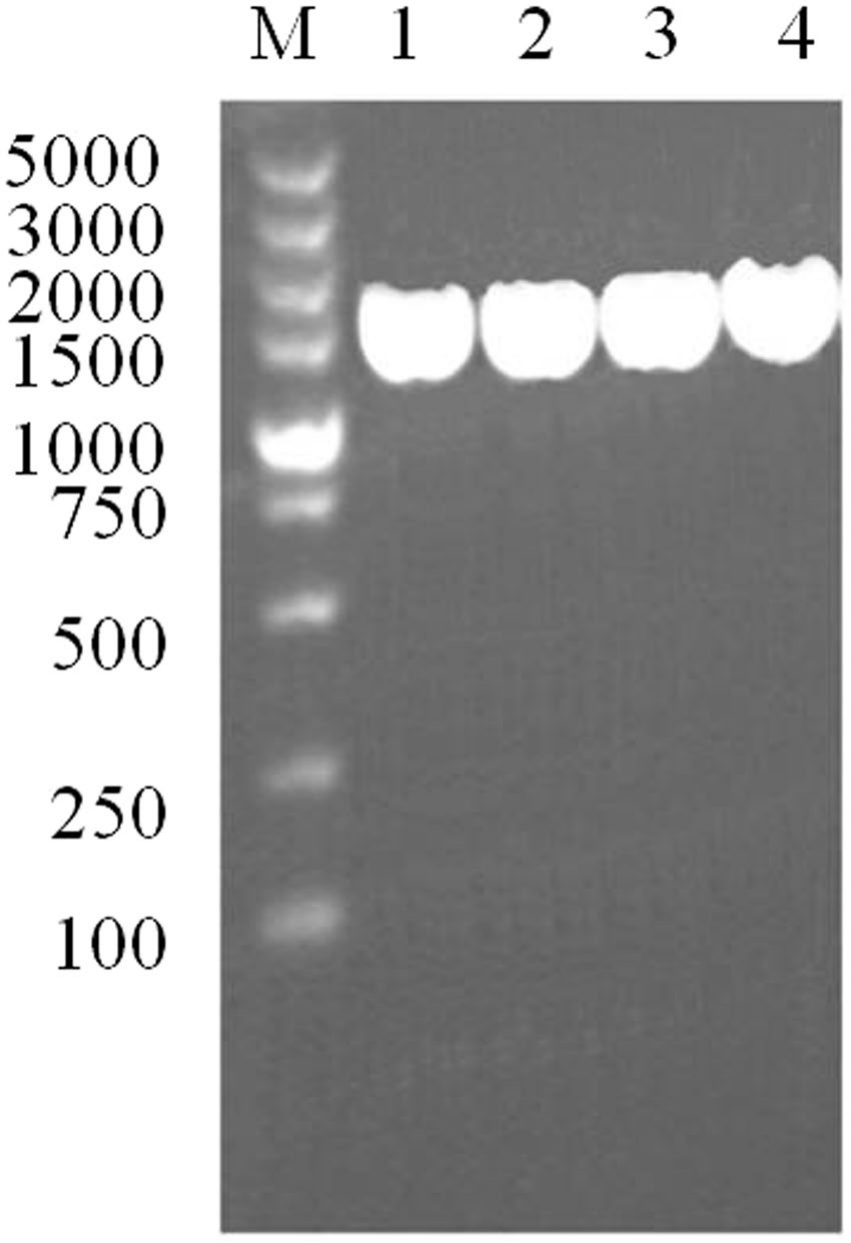
PCR products (Lane 2, 3, 4 and 5) amplified from the genomic DNA of the *P*. *polymyxa* SC-2 excreting inulinase by using the primers as described in section 2.2. Lane 1 was DNA markers; the marker sizes from top to bottom were 5.0, 3.0, 2.0, 1.5, 1.0, 0.75, 0.5, 0.25 and 0.1 kb, respectively.

**Figure 4:**
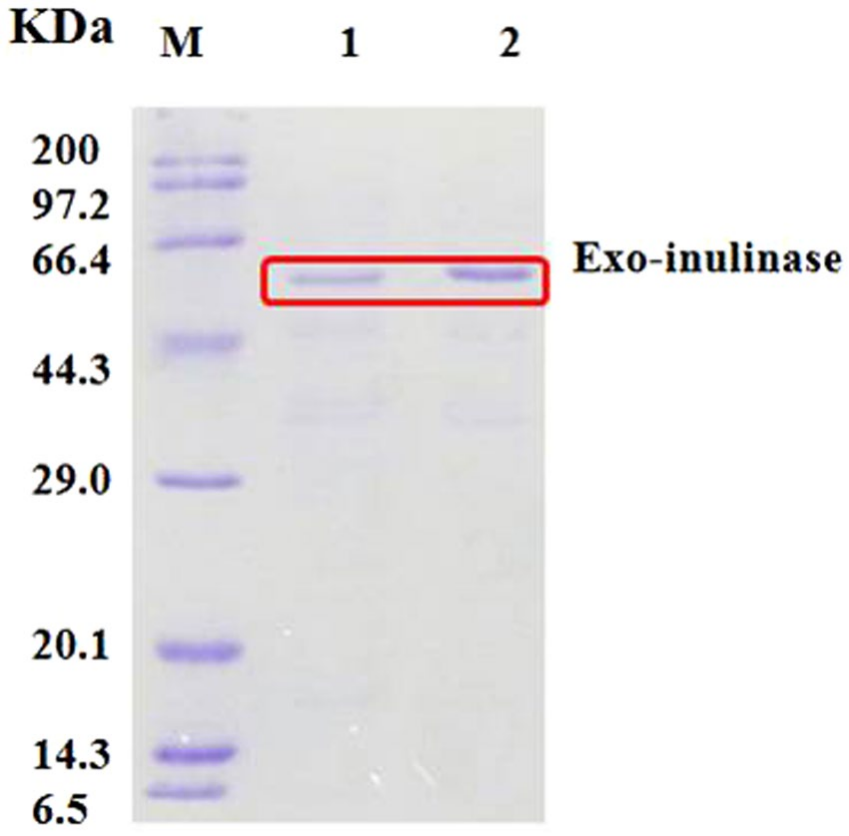
Sodium dodecyl sulfate polyacrylamide gel electrophoresis (SDS–PAGE) of the expressed recombinant inulinase. M, molecular mass standards (kDa); lane 1 and lane 2, purified exo-inulinase from the total proteins induced by isopropyl-β-D-thiogalactopyranoside (IPTG) in *C*. *tyrobutyricum*/pSY6-*inu*.

**Figure 5:**
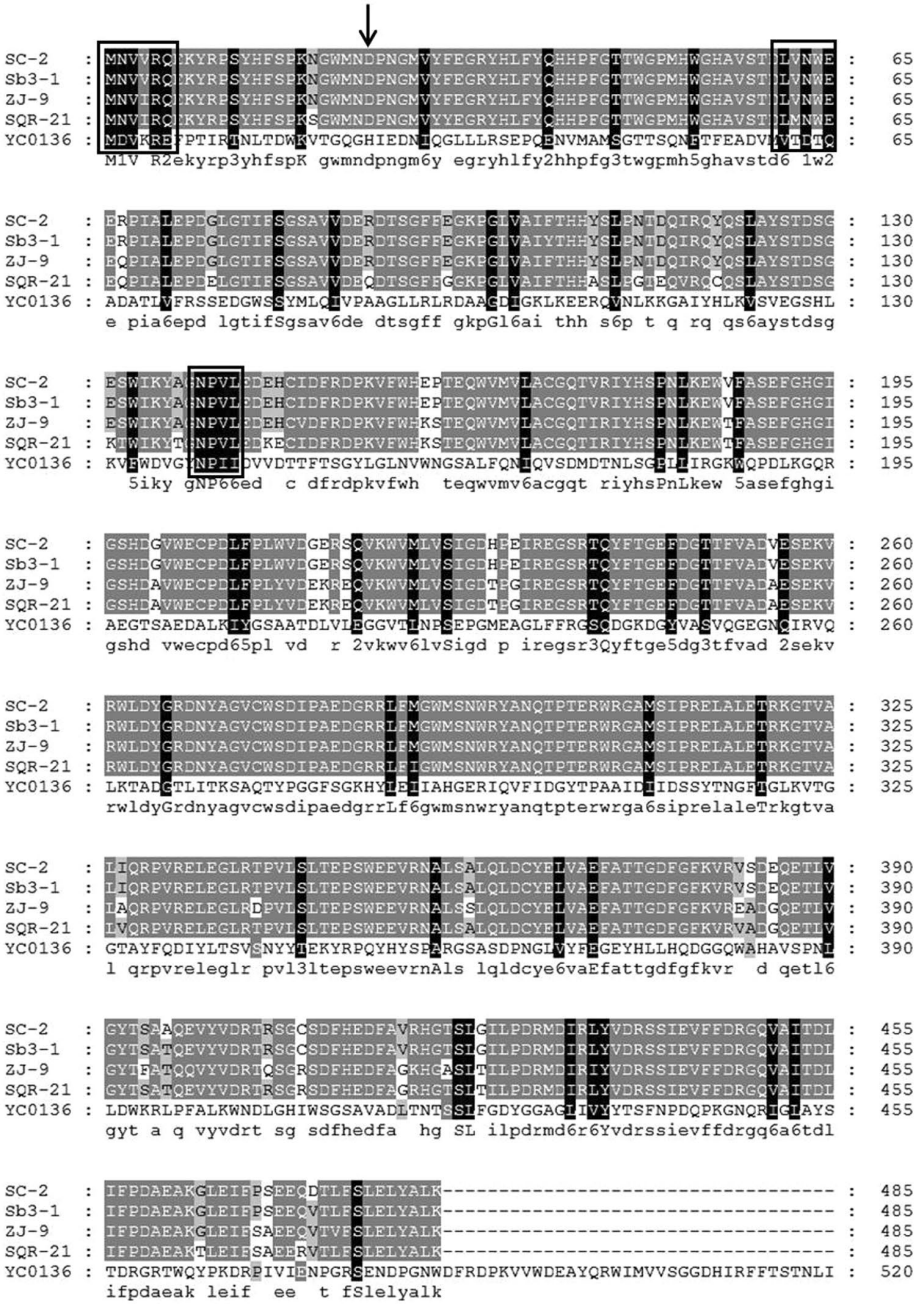
Multiple sequence alignment of inulinase from *Paenibacillus polymyxa* SC-2 (P.p SC-2), *P*. *polymyxa* ZJ-9 (P.p ZJ-9), *P*. *polymyxa* SQR-21 (P.p SQR-21), *P*. *polymyxa* Sb3-1 (P.p Sb3-1) and *P*. *polymyxa* YC0136 (P.p YC0136). The alignment was performed using NCBI database and decorated using the GenDoc software. Three highly conserved regions were found. The putative active site of glycosyl hydrolases family was marked with *downward arrows* for the sequence comparison using the PROSITE software at the Expasy website.

In order to confirm that the recombinant plasmids encompassing the exo-inulinase gene from *P*. *polymyxa* SC-2 had indeed been electro-transferred into *C*. *tyrobutyricum*, sequence analysis of the cloned *inu* gene in transformant J66 was subsequently carried out. The PCR products were obtained with the expected size, approximately 1,458 bp (Fig. 3), which encoded a protein of 485 amino acids with a calculated molecular mass of 55.5 kDa. The expressed recombinant exo-inulinase with a fused (His)_6_-tag was further purified by Ni^2+^ affinity chromatography. The purified exo-inulinase induced by IPTG exhibited an activity of 126.9 U/mL, which was 4.5-fold higher than that of the crude recombinant enzyme. The final preparation migrated as a single protein band on SDS-PAGE (Fig. 4), with a mobility corresponding to a molecular mass of about 56 kDa, which was in agreement with the predicted molecular mass of the exo-inulinase protein. A comparison of the deduced amino acid sequence of *P*. *polymyxa* SC-2 exo-inulinase with entries in the DDBJ database indicated that this enzyme is highly homologous with many Paenibacillus exo-inulinases as expected, for example, exo-inulinases from *P*. *polymyxa* Sb3-1 (sequence identity 99%), *P*. *polymyxa* ZJ-9 (93%), *P*. *polymyxa* SQR-21 (90%), and *P*. *polymyxa* YC0136 (86%) (Fig. 5). The invariability of the conserved domain in the exo-inulinase from *P*. *polymyxa* SC-2 indicates that its function in substrate binding and catalysis may not be very different from that of all known exo-acting inulinases. As expected, no such PCR products were amplified from the wild-type *C*. *tyrobutyricum* which carried no cloned exo-inulinase gene. Therefore, the transformant J66 was used for further investigations as the engineered inulin-consuming strain of *C*. *tyrobutyricum*.

### Utilization of different sugars by the engineered *C*. *tyrobutyricum* in batch SSF

Inulin typically has a degree of polymerization (DP) between 3 and 60, and chemical degradation (e.g., acid or low-pH treatment) or controlled enzymatic hydrolysis with endo-inulinases yields fructooligosaccharide compounds with a DP between 2 and 20^27^. Since wild-type *C*. *tyrobutyricum* cannot directly utilize inulin, Jerusalem artichoke need to be hydrolyzed prior to the fermentation step in order to transform inulin into available glucose and fructose^10^. In order to study the feasibility of directly using inulin as a low-cost substrate for fermentative hydrogen production, batch fermentation of inulin extract as the sole carbon source by *C*. *tyrobutyricum* mutant were studied, and compared with the two fermentable monosaccharides glucose and fructose. The total carbon source concentrations were all adjusted to 60 g/L in a 5-L fermentor system.

As shown in Table 4, *C*. *tyrobutyricum* mutant was able to utilize glucose and fructose to produce hydrogen at high yields. When the cells were grown in the fructose-containing medium, the hydrogen yield (2.8 ± 0.12 mol/mol) and overall volumetric production rate (430 ± 50 mL/h/L) were about the same as with glucose as the sole carbon source (2.6 ± 0.12 mol/mol, and 410 ± 40 mL/h/L), which was almost equivalent with that of the wild-type *C*. *tyrobutyricum* ^28^. It should be noted that the hydrogen yield from glucose or fructose depends on the fermentation end-products in *C*. *tyrobutyricum*. It has been widely accepted that the highest theoretical yield of 4 mol/mol glucose can be obtained if acetic acid is the end by-product of metabolism, while a maximum 2 mol/mol glucose can be obtained with butyric acid as the sole by-product. However, actual yields of hydrogen are always associated with a mixture of acetic and butyric acid, and in most cases, are lower than 3 mol/mol glucose^29^.

**Table 4:** Comparison of different sugars for hydrogen fermentation and cell growth of engineered *C*. *tyrobutyricum*.

Carbon sources	H_2_ Yield (mol /mol glucose)	Volumetric H_2_ production rate (mL/h/L)		Cell growth (g/L)
		Maximum	Overall^b^	
Fructose	2.8 ± 0.12	940 ± 20	430 ± 50	11.4 ± 0.87
Glucose	2.6 ± 0.16	870 ± 30	410 ± 40	10.8 ± 0.95
Inulin extract	3.5 ± 0.13^a^	1300 ± 60	540 ± 50	11.1 ± 1.09

^a^Yield of 1 mol H_2_/mol glucose is equal to 1.11 mol hydrogen/mol inulin on the basis of the stoichiometric equation.

^b^Overall volumetric H_2_ production rate = ${V}_{{H}_{2},\max }/(time\cdot rquired\cdot to\cdot reach\cdot {V}_{{H}_{2},\max }\bullet working\cdot volume)$ ${V}_{{H}_{2},\max }$: maximum cumulative hydrogen production.

Since exo-inulinase splits the terminal fructose units from inulin, it was found that the inulin extract was fermented efficiently by the engineered *C*. *tyrobutyricum*, enabling high levels of production with a yield of 3.5 ± 0.13 mol/mol inulin-type sugar and an overall volumetric production rate of 540 ± 50 mL/h/L. Monosaccharides, disaccharides, and trisaccharides were released after inulin was hydrolyzed by the inulinase expressed by the engineered cells, while only a small amount of monosaccharides and oligosaccharides could be detected after the autoclaving process (Fig. S2). This once again proved that the inulinase engineered into the *C*. *tyrobutyricum* cells was an exo-inulinase. The results of this study therefore indicate a great potential for producing hydrogen from inulin-containing materials, such as Jerusalem artichoke. In addition, the growth of mutant cells was not significantly influenced by different sugars.

### Effects of initial inulin concentration on hydrogen production in batch SSF

Bacterial growth and productivity tends to be low if high initial concentrations of reducing sugars are used in the medium due to substrate inhibition, which is a common phenomenon in practical applications^28^. A relevant characteristic of the media formulated with high initial sugar concentrations is the intrinsic high osmolarity due to the elevated amounts of media components. For instance, the osmolality of media with 120 g/L of glucose was as high as 1.8 Osm/kg in the most concentrated medium, which would inhibit the growth of saccharolytic clostridia^30^. By contrast, we used initial concentrations of 60–120 g/L of inulin extract in batch SSF mode with engineered *C*. *tyrobutyricum* (Table 5) without a problem. When the initial total sugar concentration was set at a level of 100 g/L, the highest hydrogen yield of 3.7 ± 0.22 mol/mol inulin-type sugar and overall volumetric production rate of 650 ± 70 mL/h/L were achieved. Further addition of inulin did not improve hydrogen production, but decreased both the yield and productivity due to the exhaustion of other nutrients, which was consistent with previous studies^31^.

**Table 5:** Effects of initial inulin concentration on hydrogen production of engineered *C*. *tyrobutyricum* in batch SSF.

Total sugar con. (g/L)	Total residual sugar con. (g/L)	Total amount (L)	Fermentation time (h)	Yield (mol/mol glucose)^a^	Volumetric production rate (mL/h/L)
60	0.8 ± 0.4	66.1 ± 0.05	72	3.5 ± 0.13	540 ± 50
80	1.5 ± 0.2	73.1 ± 0.05	90	3.5 ± 0.14	570 ± 80
100	6.0 ± 0.3	84.4 ± 0.06	96	3.7 ± 0.22	650 ± 70
120	10.1 ± 0.2	52.5 ± 0.04	126	2.2 ± 0.24	400 ± 30

^a^Yield of 1 mol hydrogen/mol glucose is equal to 1.11 mol hydrogen/mol inulin on the basis of the stoichiometric equation.

### Scale-up of hydrogen fermentation in batch SSF

**Figure 6:**
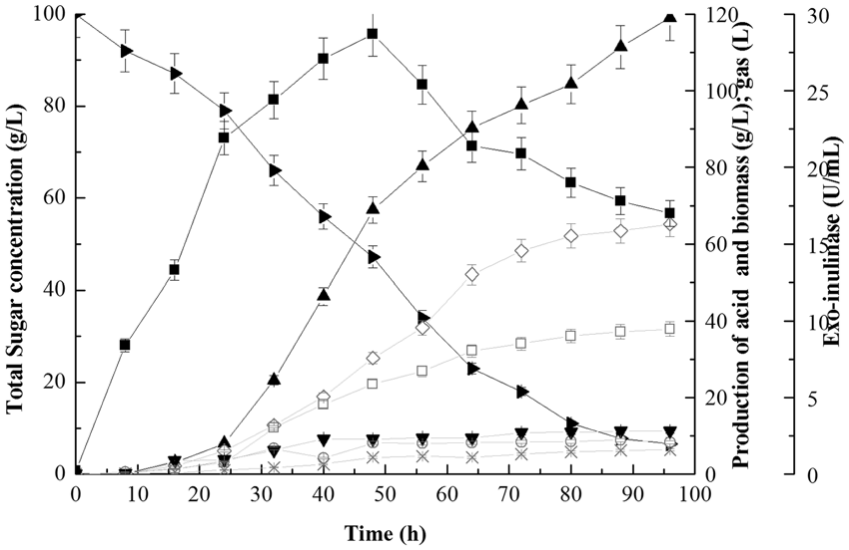
Time courses of fermentative hydrogen production by engineered *C*. *tyrobutylicum* with Jerusalem artichoke powder as the carbon source (100 g/L inulin-type sugar). For clarity, the three acid products as well as CO_2_ were all shown in grey lines. Hydrogen, ▲; Exo-inulinase, ■; Inulin, ○; Biomass, ▼; Butyric acid, □; Acetic acid, ○; Lactic acid, ×; CO_2_, ◊.

To examine the feasibility of using Jerusalem artichoke as a low-cost feedstock for fermentative hydrogen production on a pilot scale, the batch SSF of *C*. *tyrobutyricum* with 100 g/L of inulin extracted from Jerusalem artichoke as carbon source was studied in a 500-L fermentor, and the results are shown in Fig. 6. In this SSF process, the consumption of inulin started at the beginning of the fermentation, while the accumulation of free fructose was negligible throughout the fermentation. The activity of exo-inulinase was the decisive factor with regard to the conversion efficiency of inulin into reducing sugars. The time course of exo-inulinase production by *C*. *tyrobutyricum* showed that the heterologously expressed enzyme had a remarkably high inulin hydrolysis activity, reaching 28.4 ± 0.26 U/mL in the supernatant at the end of the exponential phase, which was consistent with the efficient utilization of sugar and synthesis of products. The production of hydrogen and other end-products such as organic acids (e.g., butyric, acetic and lactic acid) and gases (e.g., CO_2_) was maintained at a low level for the first 24 h, but increased afterwards and continued in the stationary phase, while the activity peak of exo-inulinase in the culture supernatant of the strain was reached after around 50 h of fermentation. The formation of hydrogen was completed at 96 h when inulin was no longer consumed. A maximum hydrogen yield of 3.5 ± 0.23 mol of H_2_ per mol of inulin-type sugar was obtained, with a volumetric productivity rate of 620 ± 60 mL/h/L based on the consumed total sugar, which was comparable to our previous results with glucose as the carbon source^29^. Importantly, the fermentative hydrogen production performance in a process model with the same experimental conditions, but with separate hydrolysis and fermentation steps (unpublished data), was much lower compared with the results of the SSF process presented in the current study. The most probable reason for this discrepancy is that the high concentration of inulin was gradually degraded to fermentable sugars in the SSF process, so that the inhibition by high substrate concentrations was avoided^32^. Therefore, SSF has an absolute competitive advantage when it comes to high initial substrate concentrations, which enables it to be operated with lower reactor volumes and therefore lower fermentation costs.

## Conclusions

Jerusalem artichoke is an abundant resource in China and is suited to serve as a cost-effective feedstock for the bio-based fermentation of value-added products. However, producing hydrogen via direct fermentation of inulin-containing materials was historically out of the question, and the necessary inulin hydrolysis was often the rate-limiting step. This study represents the first report, to our best knowledge, that inulin derived from Jerusalem artichoke was directly converted to hydrogen by an engineered *C*. *tyrobutyricum* expressing an exo-inulinase coding gene. A high hydrogen yield of 3.5 ± 0.23 mol/mol inulin-type sugar with an overall volumetric productivity rate of 620 ± 60 mL/h/L was achieved in the SSF process in a 500-L fermentator. The present study may therefore pave the way for economical bio-based hydrogen production on an industrial scale.

## Electronic supplementary material

Supplementary Information
