# Supplementary material for: Fermentative hydrogen production from Jerusalem artichoke by Clostridium tyrobutyricum expressing exo-inulinase gene
Source: Sci Rep. 2017 Aug 11;7:7940. doi: 10.1038/s41598-017-07207-7 (PMC5554141; doi:10.1038/s41598-017-07207-7)
Supplement: Supplementary file 1 — Supplementary Information [file 41598_2017_7207_MOESM1_ESM.docx]

### Fermentative hydrogen production from Jerusalem artichoke by *Clostridium tyrobutyricum* expressing exo-inulinase gene

Ling Jiang^1^, Qian Wu^2^, Qing Xu^4^, Liying Zhu^3,*^, He Huang^4,*^

^1^College of Food Science and Light Industry, ^2^College of Biotechnology and Pharmaceutical Engineering, ^3^College of Chemical and Molecular Engineering, ^4^College of Pharmaceutical Sciences, Nanjing Tech University, Nanjing 210009, People’s Republic of China

^*^Corresponding authors.

Liyng Zhu, Email: zlyhappy@njtech.edu.cn, Tel: +86-25-58139942,

College of Chemical and Molecular Engineering, Nanjing Tech University, Nanjing 210009, PR China.

He Huang, Email: biotech@njtech.edu.cn, Tel: +86-25-58139942,

College of Pharmaceutical Science, Nanjing Tech University, Nanjing 210009, PR China.


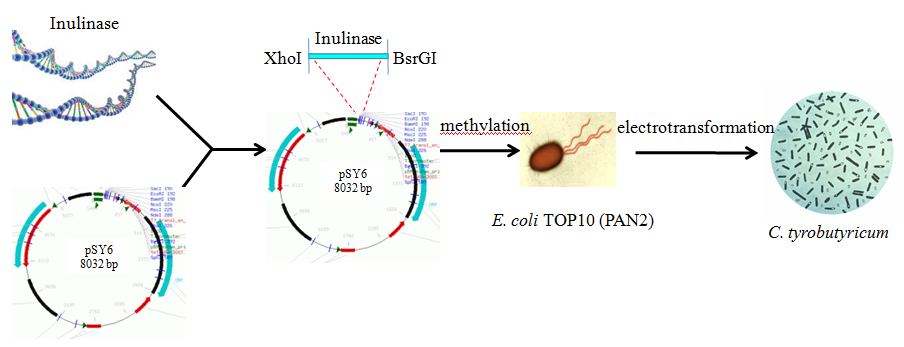


**Figure S1.** Construction of the recombinant expression vector carrying the exo-inulinase gene and transformation process.

**Figure S2.** HPLC analysis of inulin components after autoclaving pretreatment (A) and hydrolyzed by exo-inulinase expressed in *C. tyrobutyricum* mutant in 40 h of fermentation (B).
